# Evaluation of Growth and Development of Adolescents' Dental Arch Asymmetry with Normal Occlusion Using Three-Dimensional Digital Models

**DOI:** 10.1155/2021/8872022

**Published:** 2021-06-03

**Authors:** Dapeng Yang, Shiyu Ding, Peipei Li

**Affiliations:** ^1^Department of Orthodontics, Tangshan Union Medical College Hospital, Tangshan 063000, Hebei, China; ^2^Department of Stomatology, Tangshan Voccational & Technical College, Tangshan 063000, Hebei, China

## Abstract

**Objective:**

The purpose of this study was to observe the dental arch asymmetry in 12-year-olds with normal occlusion during the early permanent dentition stage.

**Methods:**

Ninety-two 12-year-old students (46 males and 46 females) who had normal occlusion during early permanent dentition were selected from a junior high school in Tangshan, China. Once per year for three consecutive years, a dental cast was obtained from each subject, and the cast was scanned with a 3D digital scanner (R700 3D). The median palatal plane (MPP) and transverse palatal plane (TPP) were used as the reference plane for the transverse and anteroposterior measurements, respectively.

**Results:**

Most of the dental arch asymmetry indicators decreased with age, but these differences were not statistically significant. The values of the midincisal edge of the upper central incisors (U1), midincisal edge of the upper lateral incisors (U2), upper canine cusp tip (U3), upper first premolar buccal cusp tip (U4), upper second premolar buccal cusp tip (U5), upper first molar mesiobuccal cusp tip (U6MB), and upper first molar distobuccal cusp tip (U6DB) to the TPP were 0.019 mm, 0.279 mm, 0.017 mm, 0.016 mm, 0.016 mm, 0.027 mm, and 0.200 mm, respectively; these values were larger in males than in females (*P* < 0.05). The values of 2–5, 6MB, and 6DB-TPP were 0.154 mm, 0.102 mm, 0.119 mm, 0.259 mm, 0.206 mm, and 0.123 mm, respectively, larger in the mandibular than in the maxillary dental arch (*P* < 0.05). The values of the midincisal edge of the lower central incisors (L1), midincisal edge of the lower lateral incisors (L2), lower canine cusp tip (L3), lower first premolar buccal cusp tip (L4), lower second premolar buccal cusp tip (L5), lower first molar mesiobuccal cusp tip (L6MB), and lower first molar distobuccal cusp tip (L6DB) to the MPP were 0.399 mm, 0.197 mm, 0.258 mm, 0.248 mm, 0.214 mm, 0.575 mm, and 0.531 mm, respectively, larger than L1-5, L6MB, and L6DB-TPP (*P* < 0.05).

**Conclusion:**

The asymmetry of the dental arch in 12-to-15-year-olds with normal occlusion did not change significantly with age. The anteroposterior asymmetry of the maxillary dental arch is larger in males than in females. With the exception of the central incisor, the anteroposterior asymmetry of the mandibular dental arch is larger than that of the maxillary dental arch. The transverse asymmetry of the mandibular dental arch is larger than the anteroposterior asymmetry.

## 1. Introduction

Maxillofacial asymmetry includes skeletal asymmetry, dental arch asymmetry, and soft tissue asymmetry [1–3]. Dental arch asymmetry has been studied extensively [4, 5] and is related to many diseases. Manfredini et al. [6] found that patients with temporomandibular joint disorder have complicated dental arch asymmetry. Mild facial asymmetry is correlated with dental arch asymmetry [7]. Dental arch asymmetry has been studied in patients with different degrees of malocclusion based on Angle's classifications. Skrinjaric et al. [8] found that the degree of fluctuation in dental arch asymmetry is highest in class III patients. Yu et al. [9] found that the mandibular deviation of class III dental arches can misdirect the teeth to the buccolingual direction, leading to dental arch asymmetry. Veli et al. [10] found that asymmetry in dental arches with class II subdivision malocclusion does not change with increasing age. Among studies of dental arch asymmetry at different ages, Kula et al. [11] found that the maxillary and mandibular dental arches of children with deep overjet are asymmetric and that the lateral and anteroposterior asymmetric dental arches are located at the position of the maxillary first permanent molars. Maurice and Kula [12] found that sagittal asymmetry in the dental arches during the mixed dentition period is more serious than anteroposterior asymmetry. Ferrario et al. [13] found slight asymmetry in the dental arches of adults and concluded that such asymmetry is normal and acceptable.

No longitudinal study has examined the asymmetry of dental arches with normal occlusion at the beginning of permanent dentition. The purpose of this study was to follow up and observe adolescents aged 12 years with normal occlusion for two consecutive years. Three-dimensional digital models were used to observe the variations in dental arch asymmetry with respect to the subjects' age and sex; the differences in maxillary and mandibular dental arch asymmetry and sagittal and anteroposterior dental arch asymmetry were studied.

## 2. Materials and Methods

In this study, 12-year-old students who had normal occlusion during the early permanent dentition stages were selected from a junior high school in Tangshan, China. The selection criteria were as follows: (1) subjects with class I molar relationship; (2) subjects with symmetrical facial contours and without obvious deformity, prominent or retracted contour of the face; (3) subjects without deformity or missing teeth; (4) subjects without history of orthodontic treatment; (5) subjects without crossbite; and (6) subjects without a history of facial trauma or changes in physical condition.

In total, 92 research subjects were included (46 males and 46 females). All dental casts were scanned by a 3D laser scanner (R7003D Dental Scanner; 3Shape A/S, Copenhagen, Denmark). The models were analyzed by a researcher using 3Shape OrthoAnalyzer analysis software (version 10.7.10).

**Figure 1:**
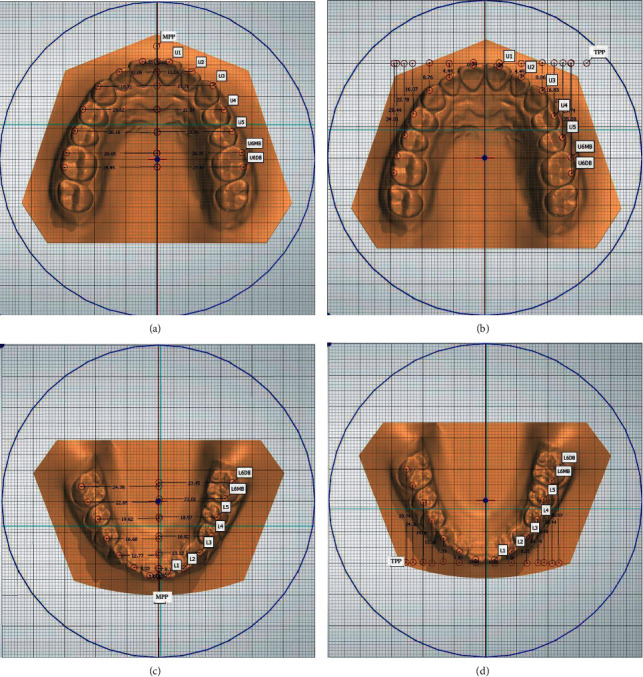
(a) Linear measurements from bilateral tooth landmarks to the MPP on the maxillary dental arch. (b) Linear measurements from bilateral tooth landmarks to the TPP on the maxillary dental arch. (c) Linear measurements from bilateral tooth landmarks to the MPP on the mandibular dental arch. (d) Linear measurements from bilateral tooth landmarks to the TPP on the mandibular dental arch.

The definitions and measurements of the mark points of these linear measurements are shown in Table 1. The reference plane for the median palatal plane (MPP) was drawn through two landmarks on the median palatal raphe. One landmark was identified as the point on the median palatal raphe adjacent to the second rugae, and the second landmark was approximately one centimeter distal to the first point on the median palatal raphe. The reference plane for the transverse palatal plane (TPP) was drawn vertical to the corresponding MPP through a common landmark (common point at the intersection of the maxillary and the mandibular dental arches (MPPB)) at the distal edges. The linear measurements of dental arch asymmetry included transverse measurements and anteroposterior measurements. Transverse measurements were taken 90° from the MPP to the bilateral landmarks. Similarly, anteroposterior measurements were taken 90° from the TPP to the bilateral landmarks. The measurements are shown in Figure 1.

The difference in distance to the MPP or TPP between the two sides was analyzed to evaluate dental arch asymmetry. To do so, the left side value was subtracted from the right side value. A positive or negative value indicates that the right dental arch is larger or smaller than the left dental arch, respectively. The mean absolute difference was calculated as the difference between two groups of bilateral MPP or TPP markers in all subjects. The value of the absolute difference indicating true severity of asymmetry was set at 2.0 mm.

Twenty cases were randomly selected and remeasured by the same examiner (Dapeng Yang) to calculate the method error. The variability of the examiner's repeated measurements was assessed by calculating the interclass correlation coefficient and measurement error for each linear distance. The mean difference of each variable between consecutive traits was compared with the paired t test. There was no significant difference between the two measurements.

## 3. Results

**Figure 2:**
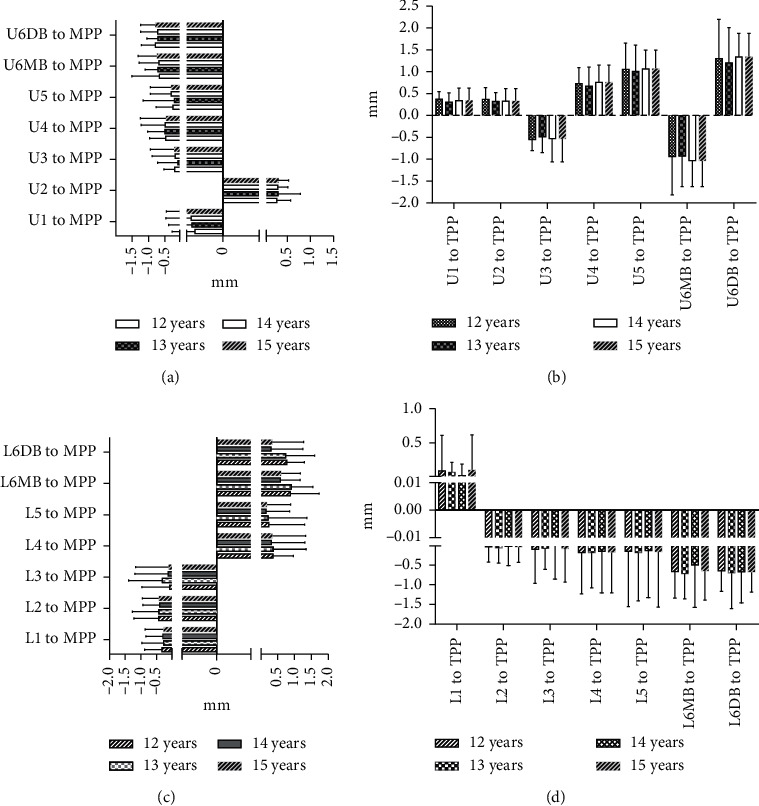
(a) Changes in the lateral asymmetry of the maxillary dental arch for two consecutive years. (b) Changes in the anteroposterior asymmetry of the maxillary dental arch for two consecutive years. (c) Changes in the lateral asymmetry of the mandibular dental arch for two consecutive years. (d) Changes in the anteroposterior asymmetry of the mandibular dental arch for two consecutive years.

**Figure 3:**
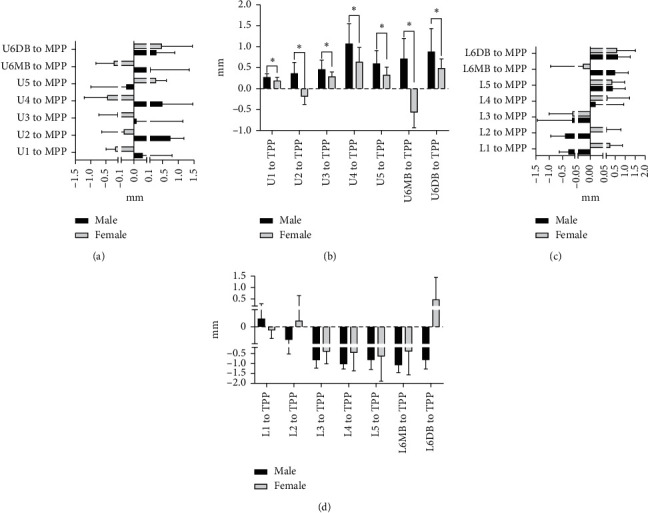
(a) Comparison of the asymmetries in the maxillary lateral arch between male and female. (b) Comparison of the asymmetries in the maxillary anteroposterior arch between male and female. (c) Comparison of the asymmetries in the mandibular lateral arch between male and female. (d) Comparison of the asymmetries in the mandibular anteroposterior arch between male and female. NS indicates nonsignificant; ^*∗*^*P*=0.05; ^*∗∗*^*P*=0.01; ^*∗∗∗*^*P*=0.001.

**Figure 4:**
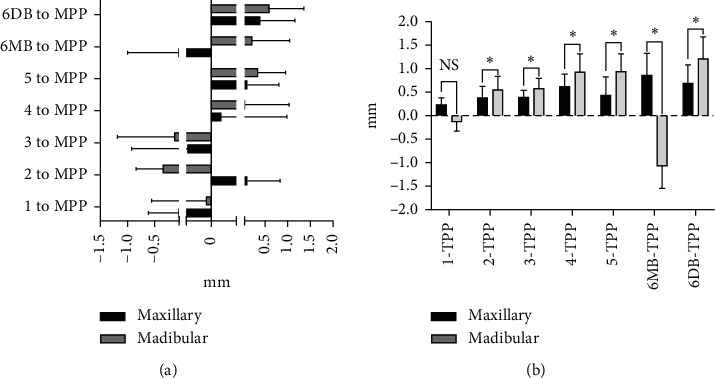
(a) Comparison of the lateral asymmetries in the maxillary and mandibular dental arches. (b) Comparison of the anteroposterior asymmetries in the maxillary and mandibular dental arches. NS indicates nonsignificant; ^*∗*^*P*=0.05; ^*∗∗*^*P*=0.01; ^*∗∗∗*^*P*=0.001.

**Figure 5:**
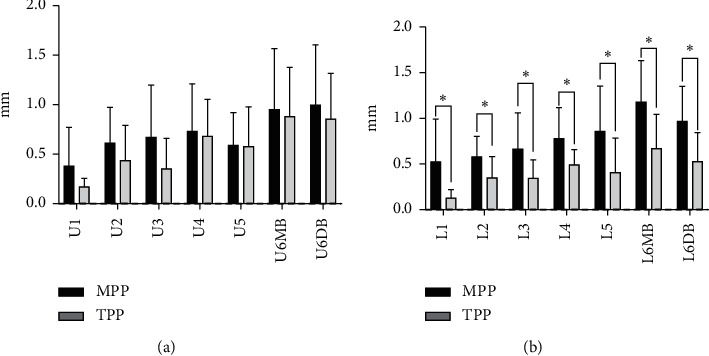
(a) Comparison of the lateral and anteroposterior asymmetries in the maxillary 257 dental arch. (b) Comparison of the lateral and anteroposterior asymmetries in the mandibular dental arch. NS indicates nonsignificant; ^*∗*^*P*=0.05; ^*∗∗*^*P*=0.01; ^*∗∗∗*^*P*=0.001.

The means and standard deviations of dental arch asymmetry studied for three consecutive years in 12-year-old adolescents with normal occlusion are summarized in Tables 2 and 3. The changes in dental arch asymmetry for three consecutive years are shown in Figure 2. The figure shows that the differences in distances from the MPP and TPP between teeth with the same name on both sides of the maxilla and mandible were less than 2 mm, indicating that there is no absolute asymmetry in the maxillary and mandibular dental arches. In addition, most of the differences decreased over three years, but not significantly. A comparison of dental arch asymmetry between males and females is shown in Figure 3. There were no differences between males and females in the values of U1–5, U6MB, and U6DB-MPP (Figure 3(a)); the values of L1–5, U6MB, and U6DB-MPP (Figure 3(c)); the values of L1-5, U6MB, and U6DB-TPP (Figure 3(d)). However, the values of U1-5, U6MB, and U6DB-TPP were 0.019 mm, 0.279 mm, 0.017 mm, 0.016 mm, 0.016 mm, 0.027 mm, and 0.200 mm, respectively, larger in males than in females (*P* < 0.05) (Table 4 and Figure 3(b)). A comparison of asymmetry between the maxillary and mandibular dental arches is shown in Figure 4. There were no differences in the values of 1–5, 6MB, and 6DB-MPP between the maxillary and mandibular dental arches (Figure 4(a)).However, the values of 2–5, 6MB, and 6DB-TPP were 0.154 mm, 0.102 mm, 0.119 mm, 0.259 mm, 0.206 mm, and 0.123 mm, respectively, larger in the mandibular dental arch than in the maxillary dental arch (*P* < 0.05) (Table 5 and Figure 4(b)).  Figure 5 illustrates the lateral and anteroposterior asymmetry of the maxillary and mandibular dental arches. There were no differences in the values of U1–5, U6MB, U6DB-MPP, U1-5, 6MB, and 6DB-TPP (Figure 5(a)). However, the values of L1–5, L6MB, and L6DB-MPP were 0.399 mm, 0.197 mm, 0.258 mm, 0.248 mm, 0.214 mm, 0.575 mm, and 0.531 mm, respectively, larger than L1–5, L6MB, and L6DB-TPP (*P* < 0.05) (Table 6 and Figure 5(b)).

## 4. Discussion

The study by Maurice [12] of the asymmetry of normal occlusion in the mixed dentition period found that dental arches are not absolutely symmetrical, but a difference of no more than 2 mm in the distances from the bilateral corresponding teeth to the reference line is considered clinically acceptable. In Figure 2(a), all values, except U2-MPP, are negative, indicating that the maxillary left dental arch is larger than the right dental arch, which is consistent with the findings of Slaj et al. [14]. However, Slaj et al. reported that dental arch symmetry in adolescents with normal occlusion deteriorated during the mixed dentition period. By contrast, Figure 2 shows that the values of most measurement indices decreased, indicating that the dental arch is symmetric; however, the decrease was not statistically significant. According to our analysis of the results, this decrease occurred because the average starting age of the subjects in this study was 12 years, which marks the beginning of permanent dentition. Dental arches tend to be stable with increasing age, and thus, the study findings are consistent with the cited literature.

Previous reports on differences in the sizes of the dental arches between sexes showed that the length, width, and height of dental arches are all larger in males than in females [15]. In terms of dental arch asymmetry, most articles in the literature conclude that there is no difference between male and female subjects [16]. However, in this study, we found that the anteroposterior asymmetry of the maxillary arches was significantly larger in males than in females. This finding is consistent with the findings of Skrinjaric et al. [8] and may be attributed to the fact that females reach peak growth and development earlier than males; therefore, their dental arches tend to stabilize earlier than males. Since the peak growth and development of women are earlier than those of men, their dental arches can be stabilized earlier, and the age of orthodontic surgery can be advanced to 16 years. The disadvantage is that women who need early orthodontic treatment should be detected and treated early by an orthodontist to avoid missing the best timing for early intervention.

De Araujo et al. [17, 18] analyzed asymmetry in dental arches with normal occlusion as belonging to Angle's class II and class I. The results showed that the asymmetry in the mandibular dental arch was larger than that in the maxillary dental arch. Nie and Jin [19] compared the asymmetry of dental arches in patients with different occlusions based on Angle's classification and found that asymmetry is larger in the mandibular dental arch than in the maxillary dental arch, except for class III occlusion cases. As shown in Figure 4, the lateral and anteroposterior asymmetry are larger in the mandibular dental arch than in the maxillary dental arch except for the position of the central incisors. In addition, the anteroposterior asymmetry is significantly larger in the mandibular dental arch than in the maxillary dental arch (Figure 4(b)). This finding is consistent with the conclusions of the cited literature.

The asymmetry of the mandibular dental arch is larger than that of the maxillary arch, which is related to not only the asymmetry of the dental arch but also the asymmetry of the jawbone. Kusayama et al. [20] investigated the relationship between lateral abnormalities of the dental arch and jawbone asymmetry and found a high correlation between dental arch abnormalities and skeletal asymmetry. Mandibular movement can increase the incidence of mandibular asymmetry but can also compensate for the asymmetry of the dental arch.

Maurice and Kula [12] found that the lateral asymmetry in dental arches is larger than the anteroposterior asymmetry. In this study, the lateral and anteroposterior asymmetries of the maxillary and mandibular dental arches were compared in detail. As shown in Figure 5(a), the differences between the distances of the corresponding teeth on both sides of the maxillary to the MPP plane were larger than the distances of the corresponding teeth on both sides of the maxillary to the TPP plane, indicating that the lateral asymmetry of the maxillary dental arch is larger than the anteroposterior asymmetry, but this difference was not statistically significant. Figure 5(b) shows that the lateral asymmetry of the mandibular dental arch is significantly larger than the anteroposterior asymmetry. This finding is related to the selected sample. The subjects in this study were teenagers with normal occlusion and Angle's class I molar relationship. Subjects with a class II or class III relationship with anteroposterior asymmetry of the dental arch were excluded. To confirm these findings, in future studies, we can select different subjects with different Angle's classifications for sagittal and anterior–posterior comparison.

## 5. Conclusions

1. The asymmetry of the dental arch in 12-to-15-year-olds with normal occlusion did not change significantly with age.
2. The anteroposterior asymmetry of the maxillary dental arch was larger in males than in females.
3. With the exception of the central incisor, the anteroposterior asymmetry of the mandibular dental arch was larger than that of the maxillary dental arch.
4. The transverse asymmetry of the mandibular dental arch is significantly larger than the anteroposterior asymmetry.

## Figures and Tables

**Table 1:** Definitions of acronyms for cast landmarks.

Acronym	Description
U1/L1	Midincisal edge of upper/lower central incisors
U2/L2	Midincisal edge of upper/lower lateral incisors
U3/L3	Upper/lower canine cusp tip
U4/L4	Upper/lower first premolar buccal cusp tip
U5/L5	Upper/lower second premolar buccal cusp tip
U6MB/L6MB	Upper/lower first molar mesiobuccal cusp tip
U6DB/L6DB	Upper/lower first molar distobuccal cusp tip
MPP	Median palatal plane
TPP	Transpalatal plane
MPPB	Common point at the intersection of the maxillary MPP and the mandibular MPP

**Table 2:** Statistical comparisons of absolute differences between horizontal and vertical measurements of maxillary bilateral dental landmarks using ordinary one-way ANOVA.

Measurement (mm)	12 years (*n* = 92)		13 years (*n* = 92)		14 years (*n* = 92)		15 years (*n* = 92)		*P*
	Mean	SD	Mean	SD	Mean	SD	Mean	SD	
U1 to MPP	−0.465	2.469	−0.252	0.371	0.725	0.452	0.725	0.652	0.548
U2 to MPP	−0.178	0.537	0.254	0.399	0.536	0.744	0.236	0.544	0.377
U3 to MPP	−0.246	0.386	−0.226	0.626	−0.148	0.774	−0.248	0.854	0.625
U4 to MPP	0.120	0.865	0.133	0.703	−0.547	1.239	−0.477	1.109	0.414
U5 to MPP	−0.282	0.298	−0.330	0.833	0.146	0.672	0.216	0.602	0.262
U6MB to MPP	−0.237	1.166	−0.317	0.576	−0.160	0.836	−0.260	0.632	0.906
U6DB to MPP	0.164	0.634	0.352	1.165	0.498	0.864	0.498	0.664	0.527
U1 to TPP	0.015	0.115	−0.129	0.475	0.068	0.155	0.048	0.175	0.318
U2 to TPP	−0.145	0.324	−0.144	0.523	−0.228	0.312	−0.228	0.841	0.526
U3 to TPP	−0.239	0.534	−0.329	0.622	0.322	0.401	0.222	0.465	0.319
U4 to TPP	−0.150	0.633	−0.594	0.670	−0.598	0.694	−0.398	0.514	0.302
U5 to TPP	−0.365	1.124	−0.598	0.213	−0.379	0.595	−0.579	0.575	0.256
U6MB to TPP	−0.363	1.183	−0.764	0.374	−0.855	0.382	−0.655	0.612	0.252
U6DB to TPP	−0.423	1.378	−0.679	0.648	−0.342	0.732	−0.442	0.732	0.446

NS indicates nonsignificant; ^*∗*^*P*=0.05; ^*∗∗*^*P*=0.01; ^*∗∗∗*^*P*=0.001.

**Table 3:** Statistical comparisons of absolute differences between horizontal and vertical measurements of mandibular bilateral dental using ordinary one-way ANOVA.

Measurement (mm)	12 years (*n* = 92)		13 years (*n* = 92)		14 years (*n* = 92)		15 years (*n* = 92)		*P*
	Mean	SD	Mean	SD	Mean	SD	Mean	SD	
L1 to MPP	−0.169	0.338	0.218	0.531	−0.111	0.344	−0.211	0.546	0.539
L2 to MPP	−0.452	0.598	0.199	0.642	−0.223	0.534	−0.243	0.544	0.376
L3 to MPP	−0.193	0.775	0.365	1.434	−0.142	1.147	−0.143	1.147	0.492
L4 to MPP	−0.288	0.716	0.377	1.152	0.260	0.952	0.161	0.872	0.121
L5 to MPP	−0.117	1.279	0.335	1.431	0.356	0.665	0.353	0.615	0.723
L6MB to MPP	0.314	1.352	0.478	1.517	0.229	0.614	0.237	0.714	0.863
L6DB to MPP	−0.182	0.553	0.403	1.339	0.627	0.551	0.617	0.451	0.544
L1 to TPP	0.115	0.527	0.176	0.131	0.129	0.262	0.129	0.142	0.784
L2 to TPP	0.192	0.445	-0.071	0.341	−0.373	0.347	−0.343	0.347	0.686
L3 to TPP	−0.122	0.748	-0.388	0.427	−0.323	0.543	−0.423	0.542	0.201
L4 to TPP	−0.168	1.539	-0.173	0.482	−0.551	0.776	−0.651	0.956	0.474
L5 to TPP	−0.632	1.197	-0.498	1.246	−0.648	0.875	−0.747	0.795	0.473
L6MB to TPP	0.273	1.436	-0.363	0.796	−0.755	0.897	−0.753	0.875	0.181
L6DB to TPP	−0.121	1.571	-0.213	1.384	−0.543	0.514	−0.513	0.954	0.332

NS indicates nonsignificant; ^*∗*^*P*=0.05; ^*∗∗*^*P*=0.01; ^*∗∗∗*^*P*=0.001.

**Table 4:** Statistical comparisons of absolute differences between male and female measurements of U1–5, U6MB, and U6DB-TPP using paired t-test analysis.

Measurement (mm)	Male (*n* = 92)		Female (*n* = 92)		*P*
	Mean	SD	Mean	SD	
U1 to TPP	0.212	0.334	0.193	0.203	^*∗*^
U2 to TPP	0.393	0.553	0.114	0.244	^*∗*^
U3 to TPP	0.312	0.293	0.295	0.267	^*∗*^
U4 to TPP	0.652	0.753	0.636	0.926	^*∗*^
U5 to TPP	0.564	0.675	0.537	0.774	^*∗*^
U6MB to TPP	0.593	1.125	0.563	0.822	^*∗*^
U6DB to TPP	0.772	1.633	0.572	0.641	^*∗*^

NS indicates nonsignificant; ^*∗*^*P*=0.05; ^*∗∗*^*P*=0.01; ^*∗∗∗*^*P*=0.001.

**Table 5:** Statistical comparisons of absolute differences between maxillary and mandibular measurements of 1–5, 6MB, 6DB-TPP using paired t-test analysis.

Measurement (mm)	Maxillary (*n* = 92)		Mandibular (*n* = 92)		*P*
	Mean	SD	Mean	SD	
1-TPP	0.253	0.34	0.141	0.653	NS
2-TPP	0.248	0.41	0.402	0.385	^*∗*^
3-TPP	0.423	0.42	0.534	0.519	^*∗*^
4-TPP	0.647	0.74	0.766	1.007	^*∗*^
5-TPP	0.454	1.044	0.713	0.855	^*∗*^
6MB-TPP	0.882	1.456	1.088	1.441	^*∗*^
6DB-TPP	0.713	1.057	0.836	1.463	^*∗*^

NS indicates nonsignificant; ^*∗*^*P*=0.05; ^*∗∗*^*P*=0.01; ^*∗∗∗*^*P*=0.001.

**Table 6:** Statistical comparisons of absolute differences between measurements of L1–5, L6MB, and L6DB to TPP and MPP using paired t-test analysis.

Measurement (mm)	MPP (*n* = 92)		TPP (*n* = 92)		*P*
	Mean	SD	Mean	SD	
L1	0.532	1.714	0.133	0.266	^*∗*^
L2	0.650	0.513	0.453	0.717	^*∗*^
L3	0.673	1.054	0.415	0.666	^*∗*^
L4	0.783	1.116	0.535	0.427	^*∗*^
L5	0.862	1.317	0.648	1.058	^*∗*^
L6MB	1.251	0.898	0.676	1.199	^*∗*^
L6DB	1.066	0.809	0.535	0.861	^*∗*^

NS indicates nonsignificant; ^*∗*^*P*=0.05; ^*∗∗*^*P*=0.01; ^*∗∗∗*^*P*=0.001.

## Data Availability

The data used to support the findings of this study are included within the article.
